# Body Composition, Physical Fitness, Physical Activity and Nutrition in Polish and Spanish Male Students of Sports Sciences: Differences and Correlations

**DOI:** 10.3390/ijerph16071148

**Published:** 2019-03-30

**Authors:** Guillermo F. López-Sánchez, Łukasz Radzimiński, Maria Skalska, Joanna Jastrzębska, Lee Smith, Dorota Wakuluk, Zbigniew Jastrzębski

**Affiliations:** 1 Faculty of Sport Sciences, University of Murcia, 30720 Murcia, Spain; 2 Department of Health Promotion, Gdansk University of Physical Education and Sport, 80-336 Gdansk, Poland; lukaszradziminski@wp.pl (Ł.R.); wkrecik@gmail.com (D.W.); zb.jastrzebski@op.pl (Z.J.); 3 Department of Pediatrics, Diabetology and Endocrinology, University Clinical Centre in Gdansk, 80-210 Gdansk, Poland; mariajastrzebska@hotmail.com; 4 Department of Pediatrics, Diabetology and Endocrinology, Gdansk Medical University, 80-210 Gdansk, Poland; joanna.jastrzebska@hotmail.com; 5 Cambridge Centre for Sport and Exercise Science, Anglia Ruskin University, Cambridge CB1 1PT, UK; Lee.Smith@anglia.ac.uk

**Keywords:** fitness, physical activity, diet, obesity, diabetes

## Abstract

It is important to study differences in body composition, physical fitness and lifestyle behaviours between university students from different countries to develop country-specific recommendations on health promotion to provide to students when transitioning to university. The present study aimed to analyse differences in body composition, physical fitness and lifestyle behaviours between Polish and Spanish students of Sports Sciences. One-hundred-and-eighty-six male students participated (81 from Poland and 105 from Spain). Polish males were on average 21.5 ± 1.9 yrs old and Spanish males 21.5 ± 2.5. The body composition variables measured were body weight (kg), fat-free mass (FFM, kg and %), fat mass (FM, kg and %), total body water (TBW, kg and %), basal metabolic rate (BMR, kcal), body mass index (BMI, kg/m^2^), fat-free mass index (FFMI, kg/m^2^) and fat mass index (FMI, kg/m^2^). The physical fitness variables measured were squat jump (SJ, height in cm, power in watts and w/kg), countermovement jump (CMJ, height in cm, power in watts and w/kg), running speed (10, 20 and 30 m (time in s)), and progressive aerobic cardiovascular endurance run (PACER, stage, final speed in km/h, distance in m, VO_2_max in mL/kg/min). Lifestyle variables measured were vigorous physical activity (VPA, days/week, min/week), moderate physical activity (MPA, days/week, min/week), walking (days/week, min/week), sitting (min/week), meals/day, vegetables/day, fruits/day, seafood/week, dairy products/week, sweets, chips, fast food/week, litres of liquid/day, litres of sugary drinks/day, alcohol/week and cigarettes/day. In comparison to Spanish students, Polish students had greater FFM (kg), greater TBW (kg), higher BMR, greater power in SJ, greater height and power in CMJ, lower times in running speed tests (10 and 20 m) and greater consumption of vegetables and liquids. In comparison to Polish students, Spanish students participated in more physical activity, and consumed more seafood, more dairy products, less sugary drinks, less alcohol and less tobacco. VPA and consumption of vegetables and liquids had positive influences on body composition and physical fitness. According to these results, universities should promote a healthy lifestyle in order to improve body composition and physical fitness in male students studying sport science. In the cases of Spain and Poland, special attention should be paid to the weak points detected in this study. This would be useful for avoiding future risk of diseases such as obesity or diabetes.

## 1. Introduction

The transition to university results in changes in lifestyle, a distancing from the family nucleus, greater independence and an increase in social relations with the peer group [1]. Many university students assume new responsibilities, becoming a vulnerable population group from the point of view of nutrition and lifestyle [2,3]. These new responsibilities can provoke a reduction of the time dedicated to do physical activity and also a decrease in the quality of diet, and as consequence of this, the body composition and physical fitness of the students can worsen considerably during the years of university [2,3]. Findings of studies conducted in several countries around the world have showed how insufficient activity, poor quality diet and smoking are serious health concerns among university students [4,5].

However, one study carried out in Spain has shown that university students of Physical Activity and Sports Sciences tend to have a healthier lifestyle, a better cardiovascular profile and less body fat than students of other university disciplines [6]. This may be owing to the curricula of Physical Activity and Sports Sciences, which promotes an active and healthy lifestyle, in addition to having practical lessons in which students learn and partake in physical activity. To the best of our knowledge no other study has been carried out investigating lifestyle behaviours and health parameters in students of Physical Activity and Sport Sciences outside of Spain. It is therefore not known whether a similar profile exists among students in other countries. 

Moreover, there are no studies comparing differences in body composition, physical fitness and lifestyle behaviours in students of Physical Activity and Sports Sciences between countries, particularly Northern and Southern Europe where there are considerable differences in the physical and social environment. It is important to understand differences in body composition and lifestyle behaviours in university students from different countries to develop country-specific recommendations on health promotion to provide to students when transitioning to university.

Therefore, the aim of this study was to analyse the differences in body composition, physical fitness and lifestyle behaviours between students of Physical Activity and Sports Sciences from two countries in Europe: Poland (Northern Europe) and Spain (Southern Europe).

## 2. Materials and Methods

### 2.1. Participants

The sample was composed of 186 male students of Physical Activity and Sports Sciences. All participants were Caucasian and were studying at university. A total of 81 males were from Poland and 105 from Spain. According to age, Polish males were on average 21.5 ± 1.9 yr old and Spanish males 21.5 ± 2.5. Inclusion criteria were being males and 3rd year Bachelor students of Sports Sciences in Gdansk (Poland) or Murcia (Spain). Exclusion criteria were having any disease or injury that does not allow participation in physical fitness tests. The participants were selected randomly from those attending the 3rd year Bachelor in Sports Sciences in Gdansk (Poland) or Murcia (Spain). The Polish 3rd year Bachelor students studied at Gdansk University of Physical Education and Sport (Poland) and the Spanish 3rd year Bachelor students at the Faculty of Sports Sciences of the University of Murcia (Spain).

Each week, Polish students participated in 22 h of lessons (10 h inactive theoretical lessons, 4 h inactive practical lessons and 8 h active practical lessons), while Spanish students participated in 21 h of lessons (11 h inactive theoretical lessons, 5 h inactive practical lessons and 5 h active practical lessons). In accordance with the Helsinki Declaration, the students were provided with detailed information about the research procedures and asked to provide written informed consent. This study received ethics approval from the research ethic committees of the institutions (Ethics code Nº UM-15-4-16).

### 2.2. Procedures

All tests were administered by the research team and research assistants with several years of experience in these tests. All tests were carried out in 2017 (February, March, April and May).

#### 2.2.1. Body Composition Analysis

All participants were measured for selected body mass and body composition variables. Measurements were performed in the morning, more than three hours after waking up and after eating and drinking. Body mass and body composition were assessed with the bioelectrical impedance method using a Tanita BC 418-MA body composition analyser (Tanita, Tokyo, Japan). All the recommendations for the analysis of bioelectrical impedance were followed. The device was calibrated to account for the weight of clothing (0.2 kg). Afterwards, data regarding age, sex and height of the subject were entered. Stadiometer HM - 250P Leicester (Marsden Scales, Rotherham, United Kingdom) was used to measure height. Then, the subjects stood on the scale with their bare feet and hands on the marked places. The device analyses body composition based on the differences of the ability to conduct electrical current by body tissues (different resistance) due to different water content. The variables measured were body weight (kg), fat-free mass (FFM, kg and %), fat mass (FM, kg and %), total body water (TBW, kg and %) and basal metabolic rate (BMR, kcal). Additionally, the following variables were calculated; body mass index (BMI, kg/m^2^), fat-free mass index (FFMI, kg/m^2^) and fat mass index (FMI, kg/m^2^). TANITA BC 418-MA is a reliable system, with good same-day test–retest reliability, no mean bias from test to test and excellent limits of agreement (< 1%). It is on a par with assessment by skinfold thickness and provides a non-invasive alternative, which requires less operator training [7].

#### 2.2.2. Jump Tests

The following vertical jumps were evaluated; Squat Jump (SJ) and Countermovement Jump (CMJ). Jump tests were carried out on an Optojump jump platform, obtaining flight time and jump height. The Optojump photocell system has a strong concurrent validity and excellent test–retest reliability for the estimation of vertical jump height: high intraclass correlation coefficients for validity (0.997–0.998), excellent test–retest reliability (0.982–0.989), low coefficients of variation (2.7%) and low random errors (±2.81 cm) [8]. To calculate peak anaerobic power output or PAPw, Sayers formula was used [9]: *PAPw (Watts) = 60.7 × jump height (cm) + 45.3 × body mass (kg)−2055.* The resulting *PAPw* obtained with this formula was divided by the body mass of the participant. 

#### 2.2.3. Running Speed Tests (10, 20 and 30 m)

In Spain running speed tests were performed on a synthetic surface (outdoor). The chosen distances were 10, 20 and 30m, starting from the standing position. Air temperature was ≈20°C, air pressure was ≈1000 mmHg and humidity was ≈60%. No precipitation occurred three days before the tests. The wind speed did not exceed 2 m/s, the starting line was always placed in the same manner to minimize the air resistance and the students ran in the direction of the wind. In Poland the tests were performed indoor on a synthetic surface. External conditions were similar to Spain. Participants carried out a 10-min warm-up to prepare the subjects for the running speed tests. The warm-up involved 5 min of running at 55% of HR_max_ and 5 min of stretching (static and dynamic). The running speed was measured for 10, 20 and 30 m runs (from a standing position). The times were measured using an electronic TAG Heuer system (model HL 2–31, Switzerland), recommended by Yeadon, Kato, & Kerwin [10], and it included two photocells with a mechanism for preventing premature switching on (starting line) and off (finishing line). This is a reliable system, with a low margin of error (<0.026 m/s) [10]. Each distance was covered by the students separately. The time between crossing the starting line and reaching the finishing line was measured. Times between other photocells within the distance were not considered. The runs were performed twice, and the shorter time results were analysed. The students always started the run from a standing position with their forward foot on the starting line. The recovery time between the tests was 5 min. Active recovery (2.5 min of marching alternated with 2.5 min of static stretching) was administered to the students during the breaks. This is a standard procedure [11,12,13].

#### 2.2.4. PACER (Progressive Aerobic Cardiovascular Endurance Run) Test

The external conditions were the same as in speed tests. This Progressive Aerobic Cardiovascular Endurance Run (PACER) (also known as beep) test is an audible, continuous and incremental maximum test until fatigue is reached. The objective of this test is to measure the maximum aerobic power. It consists of running for as long as possible between two lines with a separation of 20 m in two directions, running back and forth. In the test, the subject moves from one point to another making the change of direction to the rhythm imposed by a sound signal. The test ended when the subject reached fatigue or when he was unable to step behind the line in time on two consecutive turns. The speed obtained in the last completed stage was considered as the final speed reached and it was used to estimate the VO_2_max [14]. The reliability of PACER is very high, with a correlation between test and retest of *r* = 0.97 [11].

#### 2.2.5. Lifestyle Evaluation

Lifestyle was evaluated with a questionnaire of 14 items. All participants reported how much vigorous physical activity (VPA) and moderate physical activity (MPA) they did (days/week and minutes/week). In the questionnaire a definition of VPA and MPA was provided [15]. Participants also reported how much they walked (days/week and minutes/week) and how much time they spent sitting (mins/week). Furthermore, they reported several diet aspects: meals/day, vegetables/day, fruits/day, seafood/week, dairy products/week, sweets, chips, fast food/week, litres of liquid/day and litres of sugary drinks/day. Finally, participants also indicated how many times they drank alcohol per week and how many cigarettes they smoked per day. This questionnaire was piloted by the research team and tested in 40 students (20 Polish and 20 Spanish). During this test, the 40 students did not report any problems of understanding with any item. The questionnaire was designed according to World Health Organization (WHO) recommendations for a healthy lifestyle [15]. Physical activity recommendations for adults aged 18 to 64 years state that they should do at least 150 minutes of MPA throughout the week or do at least 75 minutes of VPA throughout the week or an equivalent combination of MPA and VPA [15]. The guidelines also state that for adults, a healthy diet should include at least 400 g (i.e., five portions) of fruit and vegetables per day; less than 10% of total energy intake from free sugars, which is equivalent to 50 g for a person of healthy body weight consuming about 2000 calories per day, but ideally is less than 5% of total energy intake for additional health benefits; less than 30% of total energy intake from fats; less than 10% of total energy intake from saturated fats, and trans-fats less than 1%; and less than 5 g of salt (equivalent to about one teaspoon) per day [15].

### 2.3. Data Analysis

A record sheet was designed in which the values obtained by each student in each of the tests were recorded. SPSS v. 23 (IBM, NY, USA) was used for all analyses and the significance was set at *p* < 0.05 in all the cases. The Kołmogorow–Smirnow test was applied to check normality, and the Levene test was used to check homogeneity of variance. Descriptive statistics were analysed and the significant differences between Polish and Spanish students were studied using the independent samples *t*-test, providing in all the variables the mean and standard deviation of each group, mean difference, *t*-value, degrees of freedom and *p*-value. In addition, the effect size was calculated using the Cohen’s *d* [16]. In the case of BMR (basal metabolic rate), analysis of covariance (ANCOVA) was used to analyse the differences between Polish and Spanish students (covariates FM % and FFM %). Furthermore, correlation analysis was carried out to study relations between lifestyle variables (physical activity and nutrition) with body composition and physical fitness. 

## 3. Results

Results cover all the participants involved in the study, as a complete data set was obtained for all participants. Regarding body composition (Table 1), Polish males had higher height, more FFM (kg), more TBW (kg) and higher BMR (kcal) than Spanish males (*p* < 0.05). When BMR was analysed taking body composition parameters as covariates, significance was maintained (*p* = 0.002). 

In physical fitness, Polish males had greater power in SJ, greater height and power in CMJ, and lower times in running speed tests of 10 m and 20 m (*p* < 0.05, Table 2). 

Spanish males had better physical activity habits than Polish males, participated in more vigorous physical activity (*p* < 0.05), more moderate physical activity (*p* < 0.05) and spent less time sitting (*p* < 0.05) (Table 3).

Spanish males consumed more seafood (*p* < 0.05), more dairy products (*p* < 0.05), less sugary drinks (*p* < 0.05), less alcohol (*p* < 0.05) and less tobacco (*p* < 0.05). Polish males consumed more vegetables (*p* < 0.05) and more liquids (*p* < 0.05) (Table 4).

In correlation analysis (Table 5 and Table 6), the three lifestyle variables with the greatest influence on body composition and physical fitness were: consumption of vegetables per day (positive influence in 14 variables), minutes of VPA per week (positive influence in 12 variables) and consumption of liquids per day (positive influence in eight variables). 

## 4. Discussion

In comparison to Spanish students, Polish students had greater FFM (kg), greater TBW (kg), higher BMR, greater power in SJ, greater height and power in CMJ, lower times in running speed tests (10 and 20 m) and more consumption of vegetables and liquids. In comparison to Polish students, Spanish students did more physical activity, and consumed more seafood, more dairy products, less sugary drinks, less alcohol and less tobacco. Moreover, VPA and consumption of vegetables and liquids had positive influences on body composition and physical fitness. Therefore, these results may be explained because, although Spanish students did more physical activity, Polish students consumed more vegetables and liquids. Another possible reason is the different university curricula (more practical lessons in Poland). Other aspect to consider is the contribution of genetic factors in physical fitness [17]. Also, Arriscado, Muros, Zabala and Dalmau [18] found inverse relationships between the percentage of body fat with maximal oxygen uptake (*r* = −0.524), lower body explosive strength (*r* = −0.400) and speed performance (*r* = 0.385), and this may explain why Polish students with more FFM (kg) have better physical fitness than Spanish students. Other studies carried out in China (*n* = 4833) also found that VO_2_max was markedly associated with FFM (*p* < 0.001) [19].

For a better understanding of these results, they can be compared with the results of other studies that analysed the same parameters in university students of other regions. Body weight in males from Gdansk was 78.80 ± 11.73 kg, in males from Murcia 75.31 ± 10.34 kg, and in other regions: male students from Madrid (Spain) 74.6 ± 6.2 kg [3], male students of Medicine from Córdoba (Spain) 75.7 ± 11.3 kg [20] and male students of Physical Education from Temuco (Chile) 73.9 ± 10.8 kg [21]. In the same way, the height of Polish students (1.80 ± 0.06 m) was higher than in males from Murcia (1.78 ± 0.06 m) and other regions: male students from Madrid (Spain) 1.78 ± 0.1 m [3], male students of Medicine from Córdoba (Spain) 1.78 ± 0.1 m [20] and male students of PE from Temuco (Chile) 1.74 ± 0.1 m [21]. These differences may be explained by genetic factors [16].

However, BMI in males from Murcia was 23.64 ± 2.50, and in males from Gdansk (24.17 ± 3.18). Polish males had 14.28 ± 5.32 % of FM, while males from Murcia 14.73 ± 5.01, and in other regions: male students from Madrid (Spain) 16.5 ± 3.5 [3], male students from Valencia (Spain) 18.75 ± 5.36 [22], male students of Medicine from Córdoba (Spain) 22.95 ± 4.48 [18] and male students of PE from Valparaíso (Chile) 22.7 ± 5.5 [23].

Regarding cardiovascular endurance, the VO_2_max of males from Gdansk was 49.86 ± 5.35 and of males from Murcia 48.64 ± 5.44, while in male students of Physical Education from Granada (Spain) it was 54.68 ± 7.16 [24]. These differences may be due to the differences in metres above sea level (Gdansk 7 m, San Javier-Murcia 4 m and Granada 738 m) and its influence on VO_2_ max [25]. 

Despite of having more FFM (kg) and better physical fitness, male students from Gdansk did less MPA (160.12 ± 166.11 min/week) and less VPA (282.69 ± 199.31 min/week) than males from Murcia, who did 318.11 ± 675.01 min/week of MPA and 285.35 ± 183.11 min/week of VPA. A possible explanation for the different levels of physical activity found could be differences in the physical environment, social and political context of the countries studied. It is also noteworthy that participants in this study were more active than university students of other degrees different to Sport Science. For example, in a systematic review [4] analysing university students’ participation in physical activity at the recommended level to acquire optimal health benefits (*n* = 35,747 students from 27 countries), it was found that more than one-half of university students were not active enough to gain health benefits, while in the present study both Polish and Spanish students of Sport Sciences did enough physical activity, according to the international recommendations [15].

With regard to tobacco, male students from Gdansk smoked 3.71 ± 5.72 cigarettes/day (39.5% were smokers), and males from Murcia 1.02 ± 2.95 cigarettes/day (16 % were smokers). These results can be compared with the study of Steptoe et al. [5], who analysed, in the year 2000, the prevalence of smoking in European university students from 13 countries: Poland 26.3%, Spain 36.3%. The smoking prevalence in the other countries varied from 23.4% in Hungary to 47.4% in Portugal [5]. These differences in lifestyle may be due to different cultures, traditions and weather in Northern and Southern Europe, but also to the implementation of efficient health policies since the year 2000.

The main strengths of the present study were the comparison between two different countries, the study of a high number of variables, and the validated tests used to evaluate body composition and physical fitness. The main limitation was that the questionnaire used to evaluate lifestyle was self-reported and not validated. This study was limited to males because there is a majority of males studying Sports Sciences in Poland and Spain. Also, as a convenience sampling was used, it was also limited to 3rd year students of Gdansk and Murcia. Therefore, our study was only representative of male 3rd year students, and our results can only be generalised to this group. Future research studies should analyse also females, students from other years and use other international samples, to have the opportunity to replicate this study with other nationalities, or even replicate it on different regions of Poland and Spain.

## 5. Conclusions

Polish male students had better values of body composition and physical fitness, while Spanish male students had healthier lifestyle. In order to avoid future risk of diseases such as obesity or diabetes, Polish curricula of Physical Activity and Sports Sciences should include more lessons that promote an active and healthy lifestyle, while Spanish curricula of Physical Activity and Sports Sciences should include more active practical lessons in which students could improve their body composition and physical fitness through physical exercise.

## Figures and Tables

**Table 1:** Comparison of body composition of males from Gdansk and Murcia.

Variables	Gdansk (*n* = 81)	Murcia (*n* = 105)	Dif.	*t*	df	Sig.	*d*
Height (m)	1.80 ± 0.06	1.78 ± 0.06	0.02	2.082	153	0.039 *	0.308
Weight (kg)	78.80 ± 11.73	75.31 ± 10.34	3.49	1.950	151	0.053	0.288
BMI (kg/m^2^)	24.17 ± 3.18	23.64 ± 2.50	0.53	1.104	117.614	0.272	0.163
FM (%)	14.28 ± 5.32	14.73 ± 5.01	0.46	0.545	151	0.587	0.081
FFM (%)	85.71 ± 5.32	85.26 ± 5.01	0.46	0.545	151	0.587	0.081
TBW (%)	62.67 ± 3.86	62.82 ± 4.93	0.15	0.206	149	0.837	0.031
FM (kg)	11.69 ± 6.05	11.41 ± 5.45	0.28	0.294	149	0.769	0.043
FFM (kg)	67.11 ± 7.13	63.92 ± 6.68	3.19	2.819	149	0.005 **	0.417
TBW (kg)	49.18 ± 5.29	47.18 ± 5.74	2.01	2.170	145	0.032 *	0.321
BMR (kcal)	1995.03 ± 232.31	1895.60 ± 182.01	99.43	2.936	148	0.004 **	0.434
FMI (kg/m^2^)	3.58 ± 1.79	3.55 ± 1.57	0.03	0.094	149	0.925	0.014
FFMI (kg/m^2^)	20.59 ± 1.74	20.10 ± 1.62	0.48	1.761	149	0.080	0.260

Values are Mean ± SD. *p* < 0.05 * *p* < 0.01 **. BMI (body mass index), FM (fat mass), FFM (fat-free mass), TBW (total body water), BMR (basal metabolic rate), FMI (fat mass index), FFMI (fat-free mass index).

**Table 2:** Comparison of physical fitness of males from Gdansk and Murcia.

Variables	Gdansk (*n* = 81)	Murcia (*n* = 105)	Dif.	*t*	df	Sig.	*d*
SJ Height (cm)	33.46 ± 4.77	31.88 ± 5.47	1.58	1.849	150	0.066	0.273
CMJ Height (cm)	41.65 ± 5.66	33.68 ± 5.80	7.97	8.442	150	0.000 **	1.248
SJ (watts)	3580.72 ± 510.73	3300.25 ± 520.95	280.47	3.177	141	0.002 **	0.469
CMJ (watts)	4064.50 ± 538.02	3410.35 ± 539.33	654.15	7.108	141	0.000 **	1.051
SJ (w/kg)	45.45 ± 3.84	43.73 ± 4.38	1.72	2.401	141	0.018 *	0.355
CMJ (w/kg)	51.69 ± 4.84	45.21 ± 4.64	6.48	8.033	141	0.000 **	1.188
Sprint 10 m (s)	1.61 ± 0.33	1.88 ±0.15	0.27	6.369	94.796	0.000 **	0.942
Sprint 20 m (s)	2.76 ± 0.58	3.19 ± 0.19	0.43	5.958	82.564	0.000 **	0.881
Sprint 30 m (s)	4.40 ± 0.19	4.43 ± 0.28	0.03	0.710	148.834	0.479	0.105
PACER (Stage)	10.09 ± 1.85	9.46 ± 1.74	0.62	1.864	114	0.065	0.276
PACER Final Speed (km/h)	12.87 ± 0.89	12.67 ± 0.90	0.20	1.207	114	0.230	0.178
PACER Distance (m)	1711.69 ± 406.30	1628.25 ± 366.38	83.44	1.162	114	0.247	0.172
PACER VO_2_max (mL/kg/min)	49.86 ± 5.35	48.64 ± 5.44	1.21	1.207	114	0.230	0.178

Values are Mean ± SD. *p* < 0.05 * *p* < 0.01 **. SJ (squat jump), CMJ (countermovement jump), PACER (Progressive Aerobic Cardiovascular Endurance Run test).

**Table 3:** Comparison of physical activity of males from Gdansk and Murcia.

Variables	Gdansk (*n* = 81)	Murcia (*n* = 105)	Dif.	*t*	df	Sig.	*d*
VPA (days/week)	3.00 ± 1.24	3.69 ± 1.53	0.69	2.460	128	0.015 *	0.364
VPA (mins/week)	282.69 ± 199.31	285.35 ± 183.11	2.66	0.074	130	0.941	0.011
MPA (days/week)	2.34 ± 1.36	3.98 ± 1.93	1.64	5.519	96.860	0.000 **	0.816
MPA (mins/week)	160.12 ± 166.11	318.11 ± 675.01	157.99	1.441	130	0.152	0.213
Walking (days/week)	4.71 ± 1.96	5.32 ± 2.23	0.61	1.418	126	0.159	0.209
Walking (mins/week)	237.56 ± 222.82	198.37 ± 224.59	39.20	0.899	127	0.371	0.133
Sitting (mins/week)	2962.56 ± 1422.28	1236.34 ± 1067.54	1726.22	7.562	125	0.000 **	1.118

Values are Mean ± SD. *p* < 0.05 * *p* < 0.01 **. VPA (vigorous physical activity), MPA (moderate physical activity).

**Table 4:** Comparison of nutrition of males from Gdansk and Murcia.

Variables	Gdansk (*n* = 81)	Murcia (*n* = 105)	Dif.	*t*	df	Sig.	*d*
Meals/day	4.10 ± 0.94	4.51 ± 0.86	0.41	2.438	130	0.016 *	0.361
Vegetables/day	2.15 ± 1.18	1.33 ± 0.94	0.82	4.222	131	0.000 **	0.624
Fruits/day	1.89 ± 1.03	1.96 ± 1.23	0.07	0.322	130	0.748	0.048
Seafood/week	0.79 ± 0.80	2.07 ± 1.19	1.28	6.140	131	0.000 **	0.908
Dairy products/week	5.61 ± 3.27	7.54 ± 5.37	1.93	2.082	131	0.039 *	0.308
Sweets, chips, fast food/week	3.35 ± 2.34	2.62 ± 1.86	0.73	1.734	58.804	0.088	0.256
Liquids (L/day)	2.81 ± 1.12	2.10 ± 0.67	0.71	3.588	46.515	0.001 **	0.531
Sugary drinks (L/day)	0.813 ± 0.78	0.394 ± 0.53	0.42	3.029	51.786	0.004 **	0.448
Alcohol/week	1.84 ± 1.71	0.73 ± 0.90	1.10	3.755	45.516	0.000 **	0.555
Cigarettes/day	3.71 ± 5.72	1.02 ± 2.95	2.69	2.754	45.209	0.008 **	0.407

Values are Mean ± SD. *p* < 0.05 * *p* < 0.01 **.

**Table 5:** Correlations physical activity—body composition and physical fitness.

Variables	VPA (Days/Week)	VPA (Mins/Week)	MPA (Days/Week)	MPA (Mins/Week)	Walking (Days/Week)	Walking (Mins/Week)	Sitting (Mins/Week)
**Height (m)**	−0.084	−0.159	−0.096	−0.099	−0.155	−0.154	0.082
**Weight (kg)**	−0.107	−0.194 *	−0.052	−0.011	−0.088	−0.155	0.002
**BMI (kg/m^2^)**	−0.084	−0.144	0.013	0.059	−0.002	−0.101	−0.057
**FM (%)**	−0.153	−0.220 *	−0.014	−0.008	−0.051	−0.176	−0.041
**FFM (%)**	0.153	0.220 *	0.014	0.008	0.051	0.176	0.041
**TBW (%)**	0.098	0.133	−0.015	0.006	0.110	0.282 **	0.054
**FM (kg)**	−0.175	−0.253 **	−0.060	−0.023	−0.064	−0.163	−0.018
**FFM (kg)**	−0.014	−0.069	−0.063	−0.002	−0.095	−0.131	0.003
**TBW (kg)**	−0.026	−0.097	−0.079	−0.009	−0.054	−0.044	0.000
**BMR (kcal)**	−0.026	−0.037	−0.025	0.002	−0.100	−0.111	0.034
**FMI (kg/m^2^)**	−0.169	−0.240 **	−0.033	−0.004	−0.039	−0.151	−0.033
**FFMI (kg/m^2^)**	0.050	0.037	0.034	0.098	0.033	−0.027	−0.075
**SJ Height (cm)**	0.045	0.259 **	−0.189 *	0.086	−0.035	0.019	−0.028
**CMJ Height (cm)**	−0.054	0.198 *	−0.243 **	0.063	−0.071	0.032	0.137
**SJ (watts)**	−0.074	−0.024	−0.191 *	0.032	−0.099	−0.143	−0.014
**CMJ (watts)**	−0.108	−0.013	−0.237 *	0.028	−0.110	−0.109	0.090
**SJ (w/kg)**	0.009	0.225 *	−0.204 *	0.081	−0.026	0.025	−0.024
**CMJ (w/kg)**	−0.054	0.192 *	−0.253 **	0.062	−0.045	0.056	0.134
**Sprint 10 m (s)**	0.060	−0.088	0.037	0.191 *	0.189	0.010	−0.110
**Sprint 20 m (s)**	0.049	−0.054	0.054	0.095	0.106	−0.004	−0.090
**Sprint 30 m (s)**	−0.035	−0.165	−0.048	0.017	0.183	−0.027	0.025
**PACER (Stage)**	0.148	0.189	−0.040	−0.122	−0.158	0.025	−0.141
**PACER Final Speed (km/h)**	0.181	0.218 *	−0.020	−0.097	−0.172	0.035	−0.146
**PACER Distance (m)**	0.191	0.213 *	−0.024	−0.109	−0.151	0.026	−0.159
**PACER VO_2_max (mL/kg/min)**	0.181	0.218 *	−0.020	−0.097	−0.172	0.035	−0.146
**SIG. CORRELATIONS**	0	12	6	1	0	1	0

Values: r (Pearson correlation coefficient). * *p* < 0.05 ** *p* < 0.01.

**Table 6:** Correlations nutrition—body composition and physical fitness.

Variables	Meals/Day	Vegetables/Day	Fruits/Day	Seafood/Week	Dairy Products/Week	Sweets/Chips/Fast Food/Week	Liquids (L/Day)	Sugary Drinks (L/Day)	Alcohol/Week	Cigarettes/Day
**Height (m)**	−0.013	0.096	−0.070	−0.080	−0.075	0.044	0.059	0.102	0.279 **	−0.049
**Weight (kg)**	−0.104	0.163	−0.224 *	0.012	−0.156	−0.048	0.129	0.111	0.095	0.011
**BMI (kg/m^2^)**	−0.114	0.146	−0.229 *	0.071	−0.157	−0.089	0.120	0.077	−0.064	0.032
**FM (%)**	−0.108	−0.046	−0.154	−0.093	−0.104	0.130	−0.071	0.075	0.122	−0.004
**FFM (%)**	0.108	0.046	0.154	0.093	0.104	−0.130	0.071	−0.075	−0.122	0.004
**TBW (%)**	0.058	0.111	0.109	0.068	0.169	−0.122	0.044	−0.089	−0.122	−0.013
**FM (kg)**	−0.148	0.033	−0.208 *	−0.079	−0.133	0.077	−0.021	0.080	0.107	0.009
**FFM (kg)**	−0.043	0.250 **	−0.175	0.050	−0.151	−0.143	0.180 *	0.103	0.061	0.009
**TBW (kg)**	−0.077	0.272 **	−0.185 *	0.035	−0.107	−0.157	0.162	0.077	0.036	−0.010
**BMR (kcal)**	−0.017	0.262 **	−0.153	0.019	−0.130	−0.114	0.186 *	0.112	0.069	0.022
**FMI (kg/m^2^)**	−0.147	0.013	−0.202 *	−0.065	−0.130	0.075	−0.030	0.074	0.069	0.003
**FFMI (kg/m^2^)**	−0.035	0.246 **	−0.163	0.174	−0.137	−0.231 *	0.201 *	0.044	−0.183 *	0.048
**SJ Height (cm)**	0.213 *	0.189 *	0.133	−0.064	−0.017	−0.028	0.108	−0.003	0.020	0.008
**CMJ Height (cm)**	0.064	0.261 **	0.055	−0.222 *	−0.093	−0.017	0.244 **	0.062	0.088	0.105
**SJ (watts)**	0.018	0.266 **	−0.151	−0.033	−0.169	−0.019	0.208 *	0.093	0.123	0.023
**CMJ (watts)**	−0.048	0.304 **	−0.162	−0.136	−0.204 *	−0.021	0.291 **	0.124	0.166	0.095
**SJ (w/kg)**	0.194 *	0.192 *	0.096	−0.070	−0.047	−0.021	0.111	0.009	0.025	0.015
**CMJ (w/kg)**	0.056	0.242 **	0.053	−0.207 *	−0.101	−0.027	0.226 *	0.057	0.099	0.123
**Sprint 10 m (s)**	−0.033	−0.044	−0.039	0.137	−0.072	−0.189 *	−0.225 *	0.030	−0.088	−0.157
**Sprint 20 m (s)**	−0.044	0.041	0.014	0.085	−0.049	−0.176	−0.115	0.006	−0.114	−0.132
**Sprint 30 m (s)**	−0.182	−0.013	−0.090	0.019	−0.130	−0.145	−0.102	0.028	−0.005	−0.054
**PACER (Stage)**	0.218 *	0.219 *	−0.046	0.186	−0.015	−0.112	−0.076	−0.017	−0.077	−0.020
**PACER Final Speed (km/h)**	0.235 *	0.211 *	−0.062	0.228 *	−0.014	−0.118	−0.074	−0.044	−0.100	−0.030
**PACER Distance (m)**	0.240 *	0.209 *	−0.041	0.226 *	−0.015	−0.109	−0.086	−0.046	−0.075	−0.024
**PACER VO_2_max (mL/kg/min)**	0.235 *	0.211 *	−0.062	0.228 *	−0.014	−0.118	−0.074	−0.044	−0.100	−0.030
**SIG. CORRELATIONS**	6	14	5	5	1	2	8	0	2	0

Values: r (Pearson correlation coefficient). * *p* < 0.05; ** *p* < 0.01.
